# Draft genome sequence of *Vibrio* sp. PBL-C16, isolated from Batu Laut Beach in Selangor, Malaysia

**DOI:** 10.1128/mra.00168-24

**Published:** 2024-06-07

**Authors:** Ummirul Mukminin Kahar, Kian Mau Goh, Iffah Izzati Zakaria, Nurfatini Radzlin, Izzatul Husna Mohd Ruslan, Suha Azizan

**Affiliations:** 1 Malaysia Genome and Vaccine Institute, National Institutes of Biotechnology Malaysia, Kajang, Selangor, Malaysia; 2 Department of Biosciences, Faculty of Science, Universiti Teknologi Malaysia, Skudai, Johor, Malaysia; 3 Department of Biochemistry, Faculty of Biotechnology and Biomolecular Sciences, Universiti Putra Malaysia, Serdang, Selangor, Malaysia; 4 Faculty of Applied Sciences, Universiti Teknologi MARA, Shah Alam, Selangor, Malaysia; 5 Department of Pharmacology, Faculty of Medicine, Universiti Malaya, Kuala Lumpur, Malaysia; The University of Arizona, Tucson, Arizona, USA

**Keywords:** *Vibrio*, genome, Batu Laut Beach, marine bacteria, glycoside hydrolase, amylase, pullulanase, chitinase, starch, pullulan

## Abstract

*Vibrio* sp. PBL-C16 is a bacterium that was isolated from Batu Laut Beach in Selangor, Malaysia. Here, we present a high-quality annotated draft genome of strain PBL-C16 and suggest its potential glycoside hydrolase enzymes for polysaccharide degradation.

## ANNOUNCEMENT

Marine bacteria were explored for their glycoside hydrolases (GHs) for many industrial applications (1). Earlier, we isolated *Celeribacter* sp. PS-C1, *Roseovarius* sp. PS-C2, and *Cellulomonas* sp. PS-H5 from Sekinchan Beach in Selangor, Malaysia (3.5029N, 101.0945E) (2–4). Genome data suggest that these bacteria harbor novel GHs for polysaccharide degradation (2–4). In this study, *Vibrio* sp. PBL-C16 was isolated from wet sediment and mud (upper 15 cm layer) of Batu Laut Beach (2.6747N, 101.5166E), 136 km away from the previous sampling site. Genomic DNA was extracted from PBL-C16 using the Monarch genomic DNA purification kit (NEB, USA), following the manufacturer's instructions. The 16S rRNA gene was amplified by PCR using 27F and 1492R primers (5), sequenced, and taxonomically identified using the NCBI and EzBioCloud 16S databases (6). The analyses revealed that PBL-C16 was most closely related (99.73%) to *Vibrio alginolyticus* NBRC 15630^T^ (GenBank accession number X56576.1). Here, we report the genome sequence of *Vibrio* sp. PBL-C16 and analyze its GHs for potential biotechnological applications.

Strain PBL-C16 was grown on marine agar (Condalab, Spain) at 40°C (pH 6.0) for 24 h. PBL-C16 genomic DNA was extracted from a single colony of the cells from a marine agar plate using the standard protocol of the Monarch genomic DNA purification kit. The library was prepared using the standard protocol of the Native Barcoding Kit 24 (SQK-NBD112.24, ONT, UK). Sequencing was performed using a Nanopore MinION MK1C instrument with an R10.4 flow cell (FLO-MIN112). The raw reads were subjected to base calling in super accurate mode and barcode trimming using the MinKNOW v22.08.4 (ONT) with Guppy v6.2.7 and aligned using minimap2 v2.22-r1101 (7). *De novo* assembly of the genome was performed using Flye v2.9 (8) with a minimum read overlap of 2,000 bp and annotated using the NCBI Prokaryotic Genome Annotation Pipeline v6.6 (9). Genome comparison between PBL-C16 and all 162 available genomes of *Vibrio* spp. in the NCBI genome database (February 2024) was performed using the average nucleotide identity (ANI) function in the EzBioCloud server (10). Carbohydrate-active enzymes (CAZymes) present in the PBL-C16 genome were mined using dbCAN3 (11). Default parameters were used for all software tools, unless otherwise specified.

The sequencer generated 248,951,423 bases from 84,092 reads (read *N_50_*, 5,847 bp). The PBL-C16 genome has a size of 5,190,524 bp, contributed by three contigs, with a coverage of 48×, and a G + C content of 44.5%. A total of 4,695 genes were identified in the genome, including 4,477 protein-coding sequences, 168 RNAs (12 5S rRNAs, 12 16S rRNAs, 12 23S rRNAs, 128 tRNAs, and four ncRNAs), and 50 pseudogenes. The genome comparison analyses showed that PBL-C16 was closely related to *Vibrio alginolyticus* NBRC 15630^T^ (ANI, 98.5%). The PBL-C16 genome encoded 153 CAZymes, including 79 GHs, 55 glycoside transferases, five polysaccharide lyases, six carbohydrate esterases, and eight auxiliary activity enzymes. PBL-C16 harbors 12 GHs belonging to GH family 13 (a pullulanase, seven α-amylases, and four chitinases) that are important for pullulan, starch, and chitin degradations. Collectively, the PBL-C16 genome provides several GH candidates for polysaccharide degradation.

## Data Availability

This Whole Genome Shotgun project has been deposited in NCBI GenBank under BioProject accession number PRJNA1049513, BioSample accession number SAMN38656959, and GenBank accession number JAXLNY000000000. The version described in this paper is the first version, JAXLNY000000000.1. The raw sequencing reads have been deposited in the NCBI Sequence Read Archive under accession number SRR27180048. The 16S rRNA gene sequence of *Vibrio* sp. PBL-C16 has been deposited in NCBI GenBank under the accession number OR898456.1.

## References

[1] Ashcroft E, Munoz-Munoz J. 2024. A review of the principles and biotechnological applications of glycoside hydrolases from extreme environments. Int J Biol Macromol 259:129227. doi:10.1016/j.ijbiomac.2024.129227 38185295

[2] Radzlin N, Yaakop AS, Goh KM, Liew KJ, Zakaria II, Kahar UM. 2022. Genome analysis of Celeribacter sp. PS-C1 isolated from Sekinchan Beach in Selangor, Malaysia, reveals its β-glucosidase and licheninase activities. Microorganisms 10:410. doi:10.3390/microorganisms10020410 35208867 PMC8874975

[3] Radzlin N, Mohd Omar S, Liew KJ, Goh KM, Zakaria II, Kahar UM. 2021. Draft genome sequence of Roseovarius sp. PS-C2, isolated from Sekinchan Beach in Selangor, Malaysia. Microbiol Resour Announc 10:e0067321. doi:10.1128/MRA.00673-21 34553998 PMC8459661

[4] Radzlin N, Low KO, Liew KJ, Goh KM, Zakaria II, Kahar UM. 2021. Draft genome sequence of Cellulomonas sp. PS-H5, isolated from Sekinchan Beach in Selangor, Malaysia. Microbiol Resour Announc 10:e0095621. doi:10.1128/MRA.00956-21 34709049 PMC8552637

[5] Chen Y-L, Lee C-C, Lin Y-L, Yin K-M, Ho C-L, Liu T. 2015. Obtaining long 16S rDNA sequences using multiple primers and its application on dioxin-containing samples. BMC Bioinformatics 16 Suppl 18:S13. doi:10.1186/1471-2105-16-S18-S13 PMC4682383 26681335

[6] Yoon S-H, Ha S-M, Kwon S, Lim J, Kim Y, Seo H, Chun J. 2017. Introducing EzBioCloud: a taxonomically united database of 16S rRNA gene sequences and whole-genome assemblies. Int J Syst Evol Microbiol 67:1613–1617. doi:10.1099/ijsem.0.001755 28005526 PMC5563544

[7] Li H. 2018. Minimap2: pairwise alignment for nucleotide sequences. Bioinformatics 34:3094–3100. doi:10.1093/bioinformatics/bty191 29750242 PMC6137996

[8] Kolmogorov M, Yuan J, Lin Y, Pevzner PA. 2019. Assembly of long, error-prone reads using repeat graphs. Nat Biotechnol 37:540–546. doi:10.1038/s41587-019-0072-8 30936562

[9] Tatusova T, DiCuccio M, Badretdin A, Chetvernin V, Nawrocki EP, Zaslavsky L, Lomsadze A, Pruitt KD, Borodovsky M, Ostell J. 2016. NCBI prokaryotic genome annotation pipeline. Nucleic Acids Res 44:6614–6624. doi:10.1093/nar/gkw569 27342282 PMC5001611

[10] Yoon S-H, Ha S-M, Lim J, Kwon S, Chun J. 2017. A large-scale evaluation of algorithms to calculate average nucleotide identity. Antonie Van Leeuwenhoek 110:1281–1286. doi:10.1007/s10482-017-0844-4 28204908

[11] Zheng J, Ge Q, Yan Y, Zhang X, Huang L, Yin Y. 2023. dbCAN3: automated carbohydrate-active enzyme and substrate annotation. Nucleic Acids Res 51:W115–W121. doi:10.1093/nar/gkad328 37125649 PMC10320055

